# *Escherichia coli* Subtilase Cytotoxin

**DOI:** 10.3390/toxins2020215

**Published:** 2010-01-28

**Authors:** Adrienne W. Paton, James C. Paton

**Affiliations:** Research Centre for Infectious Diseases, School of Molecular and Biomedical Science, University of Adelaide, Adelaide, SA 5005, Australia; Email: adrienne.paton@adelaide.edu.au

**Keywords:** subtilase cytotoxin, AB5 toxin, BiP/GRP78, endoplasmic reticulum stress, Shiga toxigenic *Escherichia coli*

## Abstract

Subtilase cytotoxin (SubAB) is the prototype of a new AB_5_ toxin family produced by a subset of Shiga toxigenic *Escherichia coli* (STEC) strains. Its A subunit is a subtilase-like serine protease and cytotoxicity for eukaryotic cells is due to a highly specific, single-site cleavage of BiP/GRP78, an essential Hsp70 family chaperone located in the endoplasmic reticulum (ER). This cleavage triggers a severe and unresolved ER stress response, ultimately triggering apoptosis. The B subunit has specificity for glycans terminating in the sialic acid *N*-glycolylneuraminic acid. Although its actual role in human disease pathogenesis is yet to be established, SubAB is lethal for mice and induces pathological features overlapping those seen in the haemolytic uraemic syndrome, a life-threatening complication of STEC infection. The toxin is also proving to be a useful tool for probing the role of BiP and ER stress in a variety of cellular functions.

## 1. Introduction

AB_5_ toxins are key virulence factors for several notorious bacterial pathogens, which collectively cause massive global morbidity and mortality, particularly amongst children in developing countries. There are three well-characterized AB_5_ toxin families, namely the Shiga toxins (Stx) produced by Shiga toxigenic *Escherichia coli* (STEC) and *Shigella dysenteriae*, cholera toxin (Ctx) and the closely related labile enterotoxins (LT) produced by *Vibrio cholerae* and enterotoxigenic *E. coli*, respectively, and pertussis toxin (Ptx) produced by *Bordetella pertussis*. AB_5_ toxins are characterized by enzymatic A subunits capable of disrupting critical host cell functions, non-covalently linked to pentameric B subunits that bind to specific glycan receptors on target eukaryotic cells triggering toxin uptake [1]. The A subunits of Stx toxins are RNA-*N*-glycosidases which cleave a specific adenine base in 28S rRNA, thereby inhibiting eukaryotic protein synthesis in the same manner as the plant toxin ricin. The A subunits of Ctx/LT and Ptx are ADP-ribosylases which modify distinct host G proteins, resulting in alteration of intracellular cAMP levels and disregulation of ion transport mechanisms [1]. Subtilase cytotoxin (SubAB) is the prototype of a fourth AB_5_ toxin family, with a mechanism of action that differs from that of the other three families [2]. Here we summarise current knowledge regarding the molecular and cellular biology, role in disease pathogenesis, and potential cell biological applications of this new toxin. 

## 2. Basic Biological Characterisation

SubAB was discovered in a strain of STEC belonging to serotype O113:H21 that caused an outbreak of haemolytic uraemic syndrome (HUS) in South Australia [3]. HUS is a life-threatening systemic complication of STEC infection, characterised by a triad of microangiopathic haemolytic anaemia, thrombocytopoenia and renal failure. These features are a direct manifestation of endothelial damage believed to be caused by Stx after absorption into the circulation from the gut lumen [4]. O113:H21 is a prominent STEC serotype frequently associated with serious human disease, and was among the first STEC serotypes to be causally associated with HUS [5]. However, unlike other prominent HUS-associated STEC serotypes such as the infamous O157:H7, O113:H21 strains lack the locus of enterocyte effacement (LEE), which encodes important accessory virulence traits promoting intestinal colonization and gastrointestinal pathology. The clinical presentation was also unusual in this particular outbreak, as the affected patients exhibited more marked neurological involvement than in previous HUS cases seen at the same hospital. This led us to hypothesise that O113:H21 and perhaps some other virulent STEC strains might produce an additional cytotoxin capable of either augmenting the effects of Stx or causing pathology in its own right. 

We then used recombinant DNA technology to demonstrate that the O113:H21 STEC genome encodes a toxin with a distinct cytopathic effect on Vero cells to that of Stx; it was maximal after three days incubation and featured rounding of cells, detachment from the substratum, and loss of viability [2]. The novel toxin operon consists of two closely linked genes, designated *subA* and *subB*, located on the O113:H21 megaplasmid pO113. The *subA* gene encodes a 347 amino acid protein with similarity to members of the Peptidase_S8 (subtilase) family of serine proteases (pfam00082.8). Its closest bacterial relative is the BA_2875 gene product of *Bacillus anthracis*. SubA contains a “catalytic triad” comprising conserved Asp, His and Ser domains characteristic of members of the subtilase family [6]. It matches consensus sequences for these domains at 11/12, 10/11 and 10/11 positions, respectively, including the known critical active site residues Asp_52_, His_89_ and Ser_272_[2]. The *subB* gene is 16 nucleotides downstream of *subA* and encodes a 141 amino acid protein with significant similarities to putative exported proteins from *Yersinia pestis* (YPO0337; 56% identity, 79% similarity over 136 amino acids) and *Salmonella* Typhi (STY1891; 50% identity, 68% similarity over 117 amino acids). STY1891 has significant similarity (30% identity over 101 amino acids) to the S2 subunit of Ptx, but there is only 18% identity between SubB and the latter [2]. 

Both *subA* and *subB* genes are required for expression of cytotoxicity in *E. coli*, and this can be abolished by mutagenesis of any one of the three critical A subunit residues referred to above. Thus, serine protease activity is fundamental to the cytotoxic mechanism. The holotoxin is highly toxic for a range of cell types and for Vero cells, less than 0.1 pg is sufficient to kill at least 50% of the monolayer in a microplate well after 3 days [2]. This is 10-100 times more potent than the specific Vero cell cytotoxicities previously reported for both major Stx toxin types [7,8,9,10,11]. However, Morinaga *et al*. [12] have reported an additional vacuolating activity of SubAB, which appeared to be due to SubB alone. This occurred at early time points, before the SubA-dependent, protease-mediated cytotoxicity became apparent, and required high toxin doses (>1 μg/mL). More recently, Lass *et al*. [13] described a similar phenotype in SubAB-treated HeLa cells, with formation of numerous vacuoles derived from elements of the ER, Golgi and probably also the mitochondria, coupled with appearance of lipid droplets in the cytoplasm. Notably, however, these changes were dependent on the proteolytic activity of SubA, as they were not seen in cells treated with a SubAB derivative with an active site Ser_272_-Ala mutation (SubA_A272_B). 

## 3. Intracellular Target and Cytotoxic Mechanism

The intracellular target of SubA was identified by subjecting toxin-treated Vero cells to proteomic analysis. A 28 kDa fragment corresponding to the *C*-terminus of BiP (GRP78) was detected in cells treated with SubAB, but not SubA_A272_B [14]. BiP is a Hsp70 family chaperone located in the endoplasmic reticulum (ER), and the toxin cleaves a di-leucine motif (Leu_416_-Leu_417_) in the hinge region connecting its N-terminal ATPase and *C*-terminal protein-binding domains. BiP’s function is to bind to exposed hydrophobic regions of nascent secretory proteins in the ER lumen *via* its *C*-terminus; subsequent release is coupled with ATP hydrolysis and facilitates folding of the proteins into their correct conformation. BiP also maintains the permeability barrier of the ER membrane, and targets terminally mis-folded proteins *via* the Sec61 apparatus for degradation by the proteasome. BiP is also the master regulator of ER stress signaling responses, and plays a crucial role in triggering the unfolded protein response (UPR). It also exhibits anti-apoptotic properties through interference with caspase activation [15,16]. Thus, disablement of BiP by SubA-mediated proteolysis would be expected to have serious consequences for cell survival. Significantly, transfected Vero cells co-expressing a SubA protease-resistant BiP derivative (Leu_416_Asp) were refractory to SubAB-mediated cytotoxicity, directly confirming the central role of BiP cleavage in the lethal mechanism [14]. This mechanism of action is unique amongst bacterial toxins. SubA also exhibits exquisite substrate specificity; BiP was the only cellular substrate identified in the proteomic screen, and high doses of purified SubA or SubAB were incapable of cleaving even the most closely related Hsp70 family chaperones *in vitro*. This is consistent with the crystal structure of SubA which reveals an unusually deep active site cleft, relative to other subtilase family proteases [14]. 

Although the causal link between cleavage of BiP by SubAB, which can occur within 15 minutes of exposure of cells to the toxin, and eventual cell death has been established beyond reasonable doubt, our understanding of the intervening molecular events is incomplete. One proven consequence of BiP cleavage is a severe ER stress response. This involves a series of changes in cellular activity aimed at alleviating the ER stress, so that the cell can restore ER homeostasis and recover [17]. ER stress responses include the UPR, which comprises transcriptional up-regulation of ER chaperones (including BiP) to boost the folding capacity of the ER, activation of proteasome-dependent ER-associated degradation (ERAD) to remove unfolded proteins from the ER lumen, and transient inhibition of translation to slow down the traffic of nascent proteins into the ER compartment that require folding [17]. In mammals, ER stress responses are triggered by activation of PKR-like ER kinase (PERK), inositol-requiring enzyme 1 (IRE1), and activating transcription factor 6 (ATF6). These three sentinel proteins span the ER membrane and their luminal domains interact with BiP. However, BiP can be competitively displaced when unfolded proteins accumulate in the ER lumen, activating the signaling pathways. Activated PERK phosphorylates eIF2α, inhibiting general protein synthesis, yet allowing translation of some mRNAs such as ATF4, a transcriptional activator for genes that ultimately assist in re-establishing ER homeostasis [18]. After release from BiP, ATF6 traffics to the Golgi and is cleaved, releasing a 50-kDa activated form, which translocates to the nucleus. Here it binds to the ER stress response element, inducing genes encoding ER chaperones such as BiP, GRP94 and protein disulphide isomerase, as well as the transcription factors C/EBP homologous protein (CHOP) and X-box-binding protein 1 (XBP1). Activation of IRE1 splices XBP1 mRNA such that it is translated into XBP1 protein, which then up-regulates genes encoding ER chaperones and the HSP40 family member P58^IPK^. P58^IPK^ provides a feedback loop by binding and inhibiting PERK, thereby relieving the eIF2α-mediated translational block [18].

We have shown that in Vero cells, treatment with SubAB activated all three ER stress signaling pathways [19]. Active PERK-dependent phosphorylation of eIF2α occurred within 30 min of toxin treatment, and correlated with inhibition of translation. Activation of IRE1 was demonstrated by splicing of XBP1 mRNA, while ATF6 activation was demonstrated by depletion of the 90-kDa un-cleaved form, and appearance of the 50-kDa cleaved form. At least for PERK and IRE1, the rapidity with which ER stress signaling responses are triggered by exposure of cells to SubAB is consistent with the hypothesis that cleavage by the toxin causes BiP to dissociate from the signaling molecules, without the need for accumulation of unfolded proteins in the ER lumen. However, ATF6 activation in response to SubAB treatment appeared to be markedly slower. Interestingly, BiP has been reported to form a stable interaction with ATF6, with dissociation requiring direct triggering mediated by an ER stress-responsive sequence in the luminal domain of ATF6 [20]. Thus, accumulation of unfolded proteins in SubAB-treated cells may be required before the ATF6 pathway is activated. Downstream consequences of BiP cleavage that were detected during the following 24 hour period included up-regulation of GRP94, ATF4, EDEM, CHOP, and GADD34. BiP itself was also up-regulated at the mRNA level, but at the protein level, it continued to be degraded by SubAB in the ER lumen, presumably preventing restoration of ER homeostasis [19]. Thus, SubAB treatment induced a severe and sustained ER stress response in Vero cells, and at 30 h, there was evidence of apoptosis, as judged by DNA fragnentation. Interestingly, SubAB-treated HeLa cells behaved slightly differently, with evidence of partial recovery of net synthesis of full length BiP after about 16 h [13]. This might be due to a shorter half-life of the toxin in the HeLa cell ER compartment, or to poorer uptake of toxin by a sub-population of HeLa cells, perhaps reflecting reduced receptor expression. The latter is consistent with the observation that at longer time points, there are two distinct sub-populations of SubAB-treated HeLa cells: those that are apoptotic/necrotic, and those that have successfully mounted UPR and survived [13]. Notwithstanding these variations, the *in vitro* findings re activation of the PERK, IRE1 and ATF6 pathways are consistent with our observation of CHOP induction in the liver, as well as evidence of apoptosis in the kidneys, spleen and liver of SubAB-treated mice [14,21]. Morinaga *et al*. [22] also reported transient inhibition of protein synthesis in SubAB-teated Vero cells due to PERK-mediated eIF2α phosphorylation; the toxin also induced cell cycle arrest in G1 phase, possibly through down-regulation of cyclin D1 due to a combination of translational inhibition and proteasomal degradation. More recently, Matsuura *et al*. [23] demonstrated SubAB-induced apoptosis in Vero cells by DNA fragmentation and annexin V labeling, as well as activation of caspase-3, caspase-7 and caspase-8; release of cytochrome c from mitochondria was also demonstrated. 

## 4. Intracellular Trafficking

B subunit-mediated binding to cognate glycan receptors on the cell surface is an essential first step in intoxication of target cells by AB_5_ toxins. This triggers internalisation and intracellular trafficking, such that the catalytic A subunit has access to its substrate. Fluorescence co-localization with sub-cellular markers established that like Stx and Ctx, SubAB is trafficked from the cell surface *via* the Golgi to the ER *via* a retrograde pathway. However, SubAB internalisation and trafficking is exclusively clathrin-dependent, whereas Stx or Ctx can also engage the lipid raft transport pathway [24]. The route through the Golgi is also distinct, with SubAB exploiting a novel p115/golgin-84-independent, COG/Rab6/COPI-dependent mechanism, and unlike Stx, retrograde transport is not dependent on the endosomal sorting nexins SNX1 and SNX2 [25]. Trafficking of the other AB_5_ toxins also differs from SubAB because their substrates are located in the cytoplasm, while that for SubAB is confined to the ER lumen. Thus, the catalytic subunits of the other toxins must also be retro-translocated across the ER membrane, by subversion of the Sec61 translocon [26,27]. Interestingly, at least for StxA, retro-translocation is believed to occur following interaction with BiP and another chaperone HEDJ/ERdj3 [27]. SubAB is also known to inhibit ERAD, presumably through reduced Sec61-mediated trafficking of substrates [13]. Thus, it is possible that SubAB-mediated BiP cleavage might interfere with entry of StxA into the cytosol, and modulate the *in vivo* consequences of Stx intoxication in patients infected with a bacterial strain producing both toxins.

## 5. Receptor Specificity

The B subunits of both Stx and Ctx bind to host cell glycolipids (Gb_3_ and GM1, respectively) [1], whereas the S2 subunit of Ptx binds to sialated glycoproteins [28]. SubB shares about 18% amino acid identity with Ptx S2 and binds to *N*-linked glycans displayed on several glycoproteins on the surface of Vero and HeLa cells, including α2β1integrin, which is known to be heavily sialated. Moreover, RNAi knock-down of β1integrin in Vero cells abrogated the vacuolating activity of the toxin [29]. Lack of involvement of glycolipid receptors is also supported by a recent *in vivo* study in knock-out mice with defects in biosynthesis of a range of glycosphingolipids and gangliosides, none of which were protected from SubAB [30]. Byres *et al*. [31] used glycan array analysis to show that SubB has a high degree of specificity for glycans terminating with α2-3-linked *N*-glycolylneuraminic acid (Neu5Gc) with little discrimination for the penultimate moiety. Roughly 20-fold weaker binding was seen with otherwise identical glycans that terminated in α2-3-linked *N*-acetylneuraminic acid (Neu5Ac), which differs by one hydroxyl group from Neu5Gc. Binding was reduced over 30-fold if the Neu5Gc linkage was changed from α23 to α2-6, and 100-fold if the terminal sialic acid was removed. This high specificity for Neu5Gc-terminating glycans is, to the best of our knowledge, unique amongst bacterial toxins [31].

Structural analysis of SubB confirmed that it forms a homopentameric ring, like CtxB and StxB. In spite of modest amino acid sequence identity (18%), there was a high degree of structural similarity with the last 100 amino acids of Ptx S2. Neu5Gc bound to a shallow pocket halfway down the sides of the SubB pentamer, whereas identical experiments using Neu5Ac failed to show any binding [31]. The Ptx S2 sialic acid binding site is also shallow and in the same location [28]. In contrast, the B subunits of Stx, Ctx and LT, whose receptors are glycolipids rather than glycoproteins, have deep receptor binding pockets located on the base of the pentamer, juxtaposed to the membrane [32,33,34]. In the SubB-Neu5Gc complex, Neu5Gc makes key interactions with the side chains of Asp_8_, Ser_12_, Glu_36_ and Tyr_78_. Neu5Gc differs from Neu5Ac by the addition of a hydroxyl on the methyl group of the *N*-Acetyl moiety, which makes additional crucial interactions with SubB; namely the extra hydroxyl points towards and interacts with Tyr_78_^OH^ and also hydrogen bonds with the main chain of Met_10_. These key interactions could not occur with Neu5Ac, thus explaining the marked preference for Neu5Gc [31]. The biological relevance of the structural analysis was confirmed by mutagenesis of key glycan-interacting residues in SubB. Mutagenesis of Ser_12_, Glu_36_ and Tyr_78_, respectively, significantly reduced cell binding and specific cytotoxicity of the respective holotoxin. Of these, the Ser_12_ mutation had the greatest impact, reducing cytotoxicity by 99.98 per cent. Importantly, mutatgenesis of Tyr_78_, which interacts only with the OH group unique to Neu5Gc, reduced cytotoxicity by 96.9 per cent [31]. 

## 6. Strain Distribution of SubAB

PCR screening of strain collections indicates that the *subAB* operon is widely distributed and is present in STEC isolates belonging to over 30 O-serogroups emanating from Australia, Japan, Europe, North America and South America [2,35,36,37,38,39,40,41,42,43,44]. Izumiya *et al*. [36] have also reported the presence of sequence variants in *subAB* (about 90 per cent identity to the published sequence) amongst the isolates from Japan. So far, *subAB* has been detected almost exclusively in LEE-negative STEC, and there appears to be an association between presence of *subAB* and STEC carrying *stx*_2_, or *stx*_1_ + *stx*_2_, rather than *stx*_1_ alone [35,36,37,38,39,40,41,42,43,44]. However, given that at least in O113:H21, the megaplasmid that carries the *subAB* operon is capable of conjugative transmission [45], there is potential for wider dissemination amongst other *E. coli* pathotypes and possibly other Enterobacteriaceae. Indeed, a very recent study has demonstrated the production of SubAB by two non-STEC *E. coli* strains, belonging to serogroups O8 and O78 [44]. These strains were isolated from children with diarrhoea in whom no other enteric pathogens were detected, providing the first evidence, albeit circumstantial, for a role for SubAB in human diarrhoea. Sequence analysis indicated the *subAB* operon in these strains was about 90% identical to that of the prototype from STEC O113:H21. Moreover, it was encoded on the chromosome, rather than on a plasmid [44]. Clearly, mass PCR screening of stool specimens from humans or animals, rather than testing specific isolates, will be needed to provide a more complete picture of the prevalence and distribution of SubAB-producing bacteria.

## 7. Pathological Features and Potential Role in Human Disease

To date, the *in vivo* effects of SubAB have only been examined in mice. Gut colonisation with recombinant *E. coli* carrying the *subAB* operon on a low copy-number plasmid did not cause obvious diarrhoea, but nevertheless resulted in dramatic weight loss (approximately 15 per cent) over a 6-day period. In contrast, mice colonised with a clone expressing the non-toxic mutant *subA*_A272_*B* operon continued to thrive. Interestingly, toxin-affected mice appeared to recover and gained weight after about 6 days, and this correlated with sero-conversion against the toxin [2]. In a separate study, immunisation with purified SubA_A272_B also protected mice from weight loss induced by subsequent colonisation with *E. coli* expressing active SubAB [46]. Interestingly, intraperitoneal injection of purified SubAB resulted in microangiopathic haemolytic anaemia, thrombocytopoenia and renal impairment in mice. This triad of features defines Stx-induced HUS in humans. There was extensive microvascular thrombosis and other histological damage in the brain, kidneys and liver, as well as dramatic splenic atrophy. Peripheral blood leukocytes were raised at 24 hours and there was also significant neutrophil infiltration in the liver, kidneys and spleen, as well as toxin-induced apoptosis at these sites [21]. Doses as low as 200 ng were lethal and survival time after intraperitoneal injection was inversely related to toxin dose [2]. SubAB may also have more subtle effects on disease pathogenesis, perhaps through immune modulation. SubAB has recently been shown to preferentially inhibit antibody secretion of immunoglobulins by activated murine B lymphocytes, leaving cytokine secretion relatively unscathed. SubAB preferentially cleaved newly synthesized BiP in these cells, and the *C*-terminal BiP fragment remained tightly bound to nascent immunoglobulin light chains, trapping them in the ER compartment [47]. SubAB may also have pro-inflammatory properties. SubAB treatment caused transient phosphorylation of Akt and activation of NF-κB in rat renal tubular epithelials cells, and this was mediated *via* the ATF6 branch of the UPR [48]. Activation of NF-κB is believed to play an important role in HUS and renal injury. However, at sub-cytotoxic concentrations, SubAB has actually been shown to inhibit LPS-mediated NF-κB activation in a murine macrophage cell line, and to protect mice from LPS-induced endotoxic lethality and experimental arthritis [49].

These findings raise the possibility that SubAB directly contributes to pathology in humans infected with strains of STEC that produce both Stx and SubAB. However, direct extrapolation from mice to humans is problematic, because of the high specificity of the toxin for receptor glycans terminating in Neu5Gc. Humans cannot synthesise this sugar because of a mutation in the *Cmah* gene, which encodes the CMP-*N*-acetylneuraminic acid hydroxylase that converts CMP-Neu5Ac to CMP-Neu5Gc. This mutation occurred about 2 million years ago after evolutionary separation of the *Hominin* lineage from the great apes [50]. This raises the possibility of human genetic hypo-susceptibility to the toxin, as uptake would be dependent on lower affinity interactions with Neu5Ac glycans. However, humans have been shown to be capable of assimilating Neu5Gc from dietary sources and incorporating it into glycoconjugates expressed on epithelial and endothelial surfaces [51]. This would enable expression of high-affinity receptors on the cell surface, thereby conferring full susceptibility to SubAB. Indeed, *in vitro* binding of SubAB to human gut epithelium and microvascular endothelium has recently been demonstrated [31]. Binding of SubAB to human tissues *in vivo* would also be facilitated by the absence of competing Neu5Gc-containing glycoproteins in human serum or intestinal mucus [31]. Lack of protective Neu5Gc-glycoproteins in serum has also been shown to account for the unexpected susceptibility of *Cmah*-knock-out mice to injected SubAB [31]. As far as dietary sources of Neu5Gc are concerned, it is ironic that the richest sources are red meat and dairy products, and these are also the commonest source of STEC contamination. This is a unique paradigm of bacterial pathogenesis, whereby humans may directly contribute to disease through dietary choices, simultaneously exposing themselves to the risk of STEC infection and sensitising their tissues to SubAB.

Of course, the hypothesis that SubAB is responsible for disease in humans cannot be tested directly, and can only be inferred by epidemiological associations. For example, is there a link between production of SubAB and severity of STEC disease in humans or animals? To date, *subAB* has been detected almost exclusively in STEC strains that are LEE-negative, but these strains have been considered to be less virulent than LEE-positive strains such as O157:H7. However, microbiological diagnostic strategies in those clinical laboratories that test stools for STEC are often tailored specifically for detection of O157:H7 strains, rather than STEC *per se*. This leads to under-detection of LEE-negative STEC as etiological agents of disease, skewing the epidemiological data, and limiting the number of LEE-negative human STEC isolates available for analysis. Nevertheless, LEE-negative strains are quite capable of causing severe disease in humans, and production of SubAB may well increase the likelihood of life-threatening complications such as HUS. Argentina has a very high incidence of STEC disease and HUS, and comprehensive microbiological investigations have shown that many of these cases are caused by LEE-negative STEC strains that also produce SubAB [43]. This may provide a sufficient number of cases to examine whether there is an association between SubAB production and disease severity. In the meantime, clinicians need to be made aware of the potential for a distinct disease spectrum in patients infected with SubAB-producing strains. 

## 8. Applications of SubAB as a Cell Biological Tool and as a Therapeutic Agent

The exquisite specificity of SubAB for BiP and its capacity abolish BiP function *in vitro* and *in vivo* make it a powerful tool for investigation the roles of BiP and ER stress in a range of cellular processes. Since BiP is essential for survival of eukaryotic cells, BiP^-/-^ cell lines and animal models are not available, and RNA knock-down approaches are not 100% efficient. In contrast, treatment with SubAB results in rapid degradation of virtually all cellular BiP in a wide variety of cell lines, effectively mimicking a knock-out phenotype. The toxin has already been used to examine the role of BiP and/or ER stress in ERAD in HeLa cells [13], modulation of T-cell activation and inflammatory responses of a variety of cell types [52,53,54,55,56] and expression and regulation of gap junction function in mesangial cells [57]. SubAB has also been employed to demonstrate the critical role of BiP in assembly and egress of cytomegalovirus from infected cells [58,59] and in production and processing of dengue virus proteins [60]. Both viruses upregulate BiP during infection, and SubAB treatment significantly reduces infectious virus release. Interestingly, defects in chaperone function, particularly those affecting ER stress responses, are now strongly implicated in cellular senescence and a range of degenerative conditions including cataracts and Parkinson’s and Alzheimer’s diseases [61]. Thus, through its capacity to rapidly and specifically abolish BiP function, SubAB may also enable *in vitro* modeling of key events in the pathogenesis of chaperonopathies. 

SubAB may also have utility as a therapeutic agent. As mentioned previously, sub-cytotoxic doses prevented LPS-induced NF-κB-mediated endotoxic lethality and experimental arthritis in mice [49]. Certain tumours also up-regulate BiP in response to ER stress induced by rapid growth, and this is a crucial anti-apoptotic mechanism. BiP up-regulation is linked to metastatic potential and resistance to chemotherapy [62]. Thus, these tumours should be particularly susceptible to SubAB. However, an appropriate targeting strategy would be required to prevent damage to normal tissues if holotoxin were to be deployed. Alternatively, the A subunit alone could be delivered *via* a more tumour-specific targeting molecule. For example, epidermal growth factor (EGF) could be used to specifically target EGF receptor- (EGFR-) positive tumours (these include melanomas, gliomas, breast, prostate, and ovarian cancers). An EGF-SubA fusion protein has recently been shown to kill EGFR-positive rat glioma and human breast and prostate cancer cells at picomolar concentrations [63]. Cell lines expressing moderate to high levels of EGFR, which is associated with invasiveness and metastatic potential, were the most susceptible. Moreover, EGF-SubA acted synergistically with drugs such as thapsigargin that induce ER stress, enabling effective deployment at concentrations well below the cytotoxicity threshold for each component. EGF-SubA has also been shown to be efficacious *in vivo*, significantly inhibiting tumour growth in mouse xenograft models of human breast and prostate cancer [63].

## 9. Conclusions

This review has summarized the substantial body of knowledge of the biological properties of SubAB that has been generated in the six or so years since its initial discovery. However, much remains to be learnt. For example, whilst we know that SubAB triggers ER stress signalling pathways, the precise molecular and cellular events whereby this leads to apoptosis remain to be elucidated. Our understanding of its role in disease in both humans and animals is also minimal. The situation is complicated by the fact that with the exception of one very recent report [44], SubAB has been detected only in strains of *E. coli* that also produce Stx, raising fascinating questions regarding the relative contributions to pathogenesis of these two highly potent AB_5_ cytotoxins. In the first instance, extensive epidemiological investigations are required to determine whether production of SubAB correlates with increased risk of severe complications of STEC disease. Secondly, it is important to determine whether Stx and SubAB act in synergism, or whether they antagonise one another. Both toxins inhibit protein synthesis; Stx acts directly by modification of eukaryotic 28S rRNA [4], while SubAB acts indirectly through activation of eIF2α, as described above. Conversely, SubAB-mediated BiP cleavage might interfere with retro-translocation of StxA into the cytosol, limiting access to its ribosomal substrate. Both toxins also have distinct effects on inflammatory signaling pathways, which may play a significant role in pathogenesis of STEC disease. Understanding these events may be very important for clinical management of affected patients.

## References

[1] Fan E., Merritt E.A., Verlinde C.L.M.J., Hol W.G.J. (2000). AB(5) toxins: Structures and inhibitor design. Curr. Opin. Struct. Biol..

[2] Paton A.W., Srimanote P., Talbot U.M., Wang H., Paton J.C. (2004). A new family of potent AB_5_ cytotoxins produced by Shiga toxigenic *Escherichia coli*. J. Exp. Med..

[3] Paton A.W., Woodrow M.C., Doyle R.M., Lanser J.A., Paton J.C. (1999). Molecular characterization of a Shiga-toxigenic *Escherichia coli* O113:H21 strain lacking *eae* responsible for a cluster of cases of hemolytic-uremic syndrome. J. Clin. Microbiol..

[4] Paton J.C., Paton A.W. (1998). Pathogenesis and diagnosis of Shiga toxin-producing *Escherichia coli* infections. Clin. Microbiol. Rev..

[5] Karmali M.A., Petric M., Lim C., Fleming P.C., Arbus G.S., Lior H. (1985). The asociation between idiopathic hemolytic uremic syndrome and infection by Verotoxin-producing *Escherichia coli*. J. Infect. Dis..

[6] Siezen R.J., Leunissen J.A.M. (1997). Subtilases: The superfamily of subtilisin-like serine proteases. Protein Sci..

[7] Noda M., Yutsudo T., Nakabayashi N., Hirayama T., Takeda Y. (1987). Purification and some properties of Shiga-like toxin from *Escherichia coli* O157:H7 that is immunologically identical to Shiga toxin. Microb. Pathog..

[8] Yutsudo T., Nakabayashi N., Hirayama T., Takeda Y. (1987). Purification and some properties of a Vero toxin from *Escherichia coli* O157:H7 that is immunologically unrelated to Shiga toxin. Microb. Pathog..

[9] Richardson S.E., Rotman T.A., Jay V., Smith C.R., Becker L.E., Petric M., Olivieri N.F., Karmali M.A. (1992). Experimental verocytotoxemia in rabbits. Infect. Immun..

[10] Lindgren S.W., Samuel J.E., Schmitt C.K., O'Brien A.D. (1994). The specific activities of Shiga-like toxin type II (SLT-II) and SLT-II-related toxins of enterohemorrhagic *Escherichia coli* differ when measured by Vero cell cytotoxicity but not by mouse lethality. Infect. Immun..

[11] Fujii J., Matsui T., Heatherly D.P., Schlegel K.H., Lobo P.I., Yutsudo T., Ciraolo G.M., Morris R.E., Obrig T. (2003). Rapid apoptosis induced by Shiga toxin in HeLa cells. Infect. Immun..

[12] Morinaga N., Yahiro K., Matsuura G., Watanabe M., Nomura F., Moss J., Noda M. (2007). Two distinct cytotoxic activities of subtilase cytotoxin produced by Shiga-toxigenic *Escherichia coli*. Infect. Immun..

[13] Lass A., Kujawa M., McConnell E., Paton A.W., Paton J.C., Wójcik C. (2008). Decreased ER-associated degradation of α-TCR induced by Grp78 depletion with the SubAB cytotoxin. Int. J. Biochem. Cell Biol..

[14] Paton A.W., Beddoe T., Thorpe C.M., Whisstock J.C., Wilce M.C.J., Rossjohn J., Talbot U.M., Paton J.C.  (2006). AB_5_ subtilase cytotoxin inactivates the endoplasmic reticulum chaperone BiP. Nature.

[15] Gething M.J. (1999). Role and regulation of the ER charperone BiP. Cell Devel. Biol..

[16] Hendershot L.M. (2004). The ER chaperone BiP is a master regulator of ER function. Mt. Sinai J. Med..

[17] Boyce M., Yuan J. (2006). Cellular response to endoplasmic reticulum stress: A matter of life or death. Cell Death Differ..

[18] Szegezdi E., Logue S.E., Gorman A.M., Samali A. (2006). Mediators of endoplasmic reticulum stress-induced apoptosis. EMBO Reports.

[19] Wolfson J., Thorpe C.M., May K.L., Paton J.C., Jandhyala D.M., Paton A.W. (2008). Subtilase cytotoxin activates PERK, ATF6 and IRE1 endoplasmic reticulum stress-signaling pathways. Cell. Microbiol..

[20] Shen J., Snapp E.L., Lippincott-Schwartz J., Prywes R. (2005). Stable binding of ATF6 to BiP in the endoplasmic reticulum stress response. Mol. Cell. Biol..

[21] Wang H., Paton J.C., Paton A.W. (2007). Pathologic changes in mice induced by subtilase cytotoxin, a potent new *Escherichia coli* AB_5_ toxin that targets the endoplasmic reticulum. J. Infect. Dis..

[22] Morinaga N., Yahiro K., Matsuura G., Moss J., Noda M. (2008). Subtilase cytotoxin, produced by Shiga-toxigenic *Escherichia coli*, transiently inhibits protein synthesis of Vero cells *via* degradation of BiP and induces cell cycle arrest at G1 by downregulation of cyclin D1. Cell. Microbiol..

[23] Matsuura G., Morinaga N., Yahiro K., Komine R., Moss J., Yoshida H., Noda M. (2009). Novel subtilase cytotoxin produced by Shiga-toxigenic *Escherichia coli* induces apoptosis in vero cells *via* mitochondrial membrane damage. Infect. Immun..

[24] Chong D.C., Paton J.C., Thorpe C.M., Paton A.W. (2008). Clathrin-dependent trafficking of subtilase cytotoxin, a novel AB_5_ toxin that targets the endoplasmic reticulum chaperone BiP. Cell. Microbiol..

[25] Smith R.D., Willett R., Kudlyk T., Pokrovskaya I., Paton A.W., Paton J.C., Lupashin V.V. (2009). The COG comples, Rab6 and COPI define a novel Golgi retrograde trafficking pathway that is exploited by SubAB toxin. Traffic.

[26] Lencer W.I., Tsai B. (2003). The intracellular voyage of cholera toxin: Going retro. Trends Biochem. Sci..

[27] Yu M., Haslam D.B. (2005). Shiga toxin is transported from the endoplasmic reticulum following interaction with the luminal chaperone HEDJ/ERdj3. Infect. Immun..

[28] Stein P.E., Boodhoo A., Armstrong G.D., Heerze L.D., Cockle S.A., Klein M.H., Read R.J. (1994). Structure of a pertussis toxin-sugar complex as a model for receptor binding. Nature Struct. Biol..

[29] Yahiro K., Morinaga N., Satoh M., Matsuura G., Tomonaga T., Nomura F., Moss J., Noda M. (2006). Identification and characterization of receptors for vacuolating activity of subtilase cytotoxin. Mol. Microbiol..

[30] Kondo Y., Tokuda N., Fan X., Yamashita T., Honke K., Takematsu H., Togayachi A., Ohta M., Kotzusumi Y., Narimatsu H., Tajima O., Furukawa K., Furukawa K.  (2009). Glycosphingolipids are not pivotal receptors for Subtilase cytotoxin *in vivo*: Sensitivity analysis with glycosylation-defective mutant mice. Biochem. Biophys. Res. Commun..

[31] Byres E., Paton A.W., Paton J.C., Löfling J.C., Smith D.F., Wilce M.C.J., Talbot U.M., Chong D.C., Yu H., Huang S., Chen X., Varki N.M., Varki A., Rossjohn J., Beddoe T. (2008). Incorporation of a non-human glycan mediates human susceptibility to a bacterial toxin. Nature.

[32] Merritt E.A., Sarfarty S., Jobling M.G., Chang T., Holmes R.K., Hirst T.R., Hol W.G. (1997). Structural studies of receptor binding by cholera toxin mutants. Protein Sci..

[33] Merritt E.A., Sixma T.K., Kalk K.H., van Zanten B.A., Hol W.G. (1994). Galactose-binding site in *Escherichia coli* heat-labile enterotoxin (LT) and cholera toxin (CT). Mol. Microbiol..

[34] Ling H., Bast D., Brunton J.L., Read R.J. (1998). Structure of the Shiga-like toxin I B-pentamer complexed with an analogue of its receptor Gb3. Biochemistry.

[35] Paton A.W., Paton J.C. (2005). Multiplex PCR for direct detection of Shiga toxigenic *Escherichia coli* producing the novel subtilase cytotoxin. J. Clin. Microbiol..

[36] Izumiya H., Iyoda S., Terajima J., Ohnishi M., Yamasaki S., Watanabe H. (2006). Distribution of the *subA* gene among LEE-negative STEC isolates in Japan. Abstracts of the Sixth International Symposium on Shiga toxin (Verocytotoxin) -Producing Escherichia coli Infections (VTEC2006).

[37] Khaitan A., Jandhyala D.M., Thorpe C.M., Ritchie J.M., Paton A.W. (2007). The operon encoding SubAB a novel cytotoxin is present in Shiga toxin-producing *Escherichia coli* isolates from the United States. J. Clin. Microbiol..

[38] Cergole-Novella M.C., Nishimura L.S., dos Santos L.F., Irino K., Vaz T.M.I., Bergamini A.M.M., Guth B.E.C. (2008). Distribution of virulence profiles related to new toxins and putative adhesins in Shiga toxin-producing *Escherichia coli* isolated from diverse sources in Brazil. FEMS Microbiol. Lett..

[39] Wolfson J.J., Jandhyala D.M., Gorczyca L.A., Qadeer Z., Manning S.D., Hadler J., Rudrik J.T., Thorpe C.M. (2009). Prevalence of the operon encoding subtilase cytotoxin in non-O157 Shiga toxin-producing *Escherichia coli* isolated from humans in the United States. J. Clin. Microbiol..

[40] Slanec T., Fruth A., Creuzburg K., Schmidt H. (2009). Molecular analysis of virulence profiles and Shiga toxin genes in food-borne Shiga toxin-producing *Escherichia coli*. Appl. Environ. Microbiol..

[41] Karama M., Johnson R.P., Holtslander R., McEwen S.A., Gyles C.L. (2008). Prevalence and characterization of verotoxin-producing *Escherichia coli* (VTEC) in cattle from an Ontario abattoir. Can. J. Vet. Res..

[42] Irino K., Vieira M.A.M., Gomes T.A.T., Naves Z.V.F., Guth B.E.C., Oliveira M.G., Timm C.D., Pigatto C.P., Farah S.M.S.S., Vaz T.M.I. (2009). The *subAB* operon encoding the subtilase cytotoxin (SubAB) is present only among Shiga toxin-producing *Escherichia coli*. Abstracts of the Seventh International Symposium on Shiga toxin (Verocytotoxin)-Producing Escherichia coli Infections (VTEC2009).

[43] Gerhardt E., Miliwebsky E., Rivas M., Ibarra C. (2009). Detection of subtilase cytotoxin (SubAB) in Shiga toxin-producing *Escherichia coli*strains associated with hemolytic uremic syndrome in Argentine children. Abstracts of the Seventh International Symposium on Shiga toxin (Verocytotoxin)-Producing Escherichia coli Infections (VTEC2009).

[44] Tozzoli R., Caprioli A., Cappannella S., Michelacci V., Marziano M.L., Morabito S. (2010). Production of the subtilase AB5 cytotoxin by Shiga toxin-negative *Escherichia coli*. J. Clin. Microbiol..

[45] Srimanote P., Paton A.W., Paton J.C. (2002). Characterization of a novel type IV pilus locus carried on the large plasmid of human-virulent strains of locus of enterocyte effacement-negative Shiga-toxigenic *Escherichia coli*. Infect. Immun..

[46] Talbot U.M., Paton J.C., Paton A.W. (2005). Protective immunization of mice with an active site mutant of subtilase cytotoxin of Shiga toxigenic *Escherichia coli*. Infect. Immun..

[47] Hu C.A., Dougan S.K., Winter S.V., Paton A.W., Paton J.C., Ploegh H.L. (2009). Subtilase cytotoxin cleaves newly synthesised BiP and blocks antibody secretion in B lymphocytes. J. Exp. Med..

[48] Yamazaki H., Hiramatsu N., Hayakawa K., Okamura M., Huang T., Nakajima S., Yao J., Paton A.W., Paton J.C., Kitamura M. (2009). Activation of the Akt-NF-κB pathway by subtilase cytotoxin through the ATF6 branch of the unfolded protein response. J. Immunol..

[49] Harama D., Koyama K., Mukai M., Shimokawa N., Miyata M., Nakamura Y., Ohnuma Y., Ogawa H., Matsuoka S., Paton A.W., Paton J.C., Kitamura M., Nakao A. (2009). A sub-cytotoxic dose of subtilase cytotoxin prevents LPS-induced inflammatory responses, depending on its capacity to induce the unfolded protein response. J. Immunol..

[50] Varki A. (2001). Loss of N-glycolylneuraminic acid in humans: Mechanisms, consequences, and implications for hominid evolution. Am. J. Phys. Anthropol..

[51] Tangvoranuntakul P., Gagneux P., Diaz S., Bardor M., Varki N., Varki A., Muchmore E. (2003). Human uptake and incorporation of an immunogenic nonhuman dietary sialic acid. Proc. Natl. Acad. Sci. USA.

[52] Takano Y., Hiramatsu N., Okamura M., Hayakawa K., Shimada T., Kasai A., Yokouchi M., Shitamura A., Yao J., Paton A.W., Paton J.C., Kitamura M. (2007). Suppression of cytokine responses by GATA inhibitor K-7174: Implication for unfolded protein response. Biochem. Biophys. Res. Comm..

[53] Hayakawa K., Hiramatsu N., Okamura M., Yao J., Paton A.W., Paton J.C., Kitamura M. (2008). Blunted activation of NF-κB and NF-κB-dependent gene expression by geranylgeranylacetone: Involvement of unfolded protein response. Biochem. Biophys. Res. Comm..

[54] Hayakawa K., Hiramatsu N., Okamura M., Yamazaki H., Nakajima S., Yao J., Paton A.W., Paton J.C., Kitamura M. (2009). Acquisition of anergy to proinflammatory cytokines in nonimmune cells through endoplasmic reticulum stress response: A mechanism for subsidence of inflammation. J. Immunol..

[55] Du S., Hiramatsu N., Hayakawa K., Kasai A., Okamura M., Huang T., Yao J., Takeda M., Araki I., Sawada N., Paton A.W., Paton J.C., Kitamura M. (2009). Suppression of NF-κB by cyclosporin A and tacrolimus (FK506) *via* induction of the C/EBP family: Implication for unfolded protein response. J. Immunol..

[56] Okamura M., Takano Y., Hiramatsu N., Hayakawa K., Yao J., Paton A.W., Paton J.C., Kitamura M. (2009). Suppression of cytokine responses by indomethacin in podocytes: A mechanism through induction of unfolded protein response. Am. J. Physiol. Renal Physiol..

[57] Huang T., Wan Y., Zhu Y., Fang X., Hiramatsu N., Hayakawa K., Paton A.W., Paton J.C., Kitamura M., Yao J. (2009). Downregulation of gap junction expression and function by endoplasmic reticulum stress. J. Cell Biochem..

[58] Buchkovich N.J., Maguire T.G., Yu Y., Paton A.W., Paton J.C., Alwine J.C. (2008). Human Cytomegalovirus specifically controls the levels of the endoplasmic reticulum chaperone BiP/GRP78, which is required for virion assembly. J. Virol..

[59] Buchkovich N.J., Maguire T.G., Paton A.W., Paton J.C., Alwine J.C. (2009). The endoplasmic reticulum chaperone BiP/GRP78 is important in the structure and function of the HCMV assembly compartment. J. Virol..

[60] Wati S., Soo M.L., Zilm P., Li P., Paton A.W., Burrell C.J., Beard M., Carr J.M. (2009). Dengue virus infection induces GRP78 which acts to chaperone viral antigen production. J. Virol..

[61] Macario A.J.L., Conway de Macario E. (2005). Sick chaperones, cellular stress and disease. N. Engl. J. Med..

[62] Li J., Lee A.S. (2006). Stress induction of GRP78/BiP and its role in cancer. Curr. Mol. Med..

[63] Backer J.M., Krivoshein A., Hamby C.V., Pizzonia J., Gilbert K., Ray Y.S., Brand H., Paton A.W., Paton J.C., Backer M.V. (2009). Chaperone-targeting cytotoxin and ER stress-inducing drug synergize to kill cancer cells. Neoplasia.

